# Supplementary figures and images for: Host-pathogen protein interaction studies: quality control of cDNA libraries using nanopore sequencing
Source: PLoS One. 2025 May 30;20(5):e0324917. doi: 10.1371/journal.pone.0324917 (PMC12124543; doi:10.1371/journal.pone.0324917)

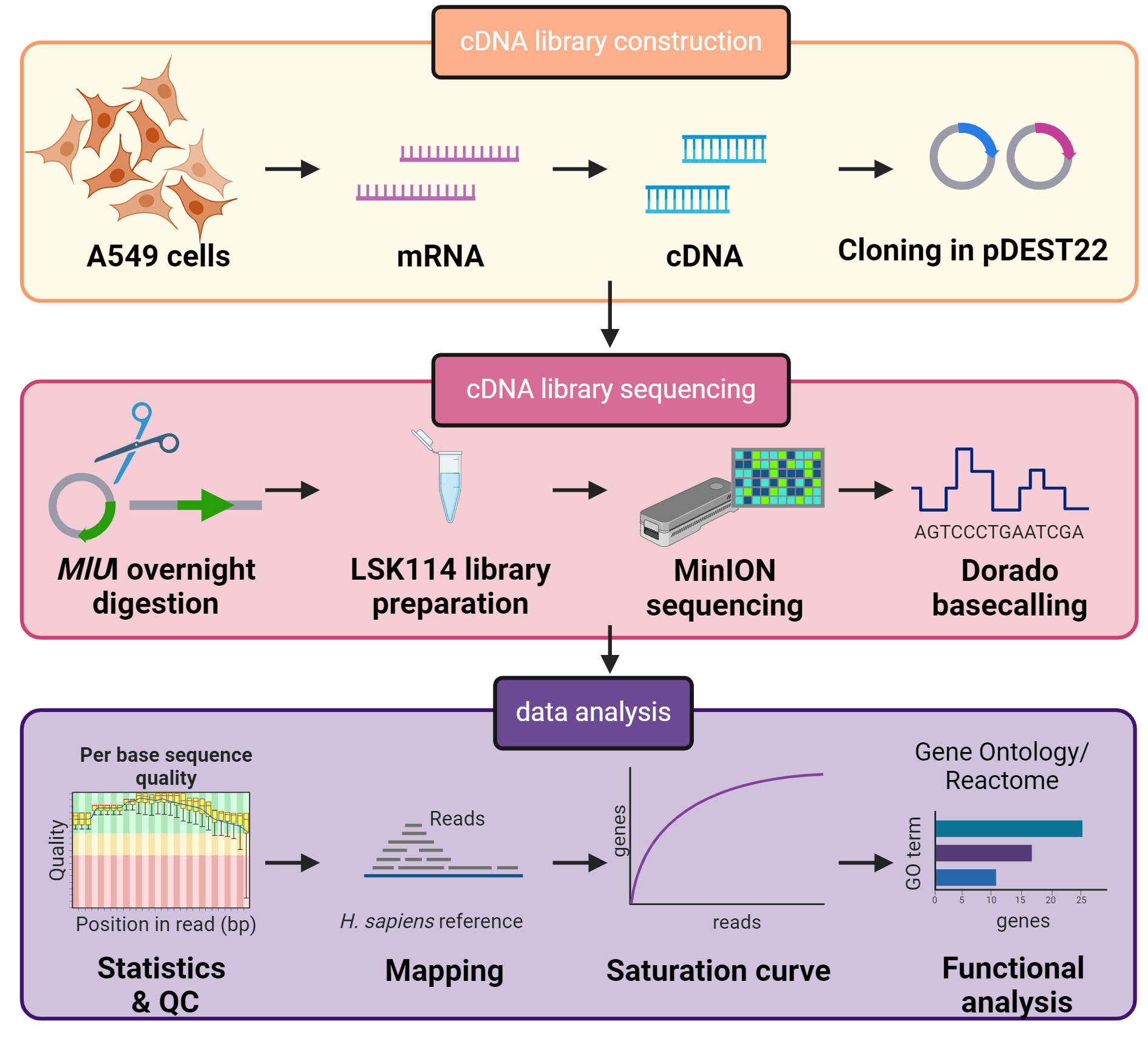

Supplement: Fig S1 — (TIF) [file pone.0324917.s001.tif]
