# Supplementary material for: Host-pathogen protein interaction studies: quality control of cDNA libraries using nanopore sequencing
Source: PLoS One. 2025 May 30;20(5):e0324917. doi: 10.1371/journal.pone.0324917 (PMC12124543; doi:10.1371/journal.pone.0324917)
Supplement: Attachment — Submitted filename: comment.docx [file pone.0324917.s011.docx]

This study innovatively applies Oxford Nanopore Technologies long-read sequencing to quality control of yeast two-hybrid (Y2H) cDNA libraries, addressing the limitations of conventional methods in assessing library coverage and functional representation. The experimental design integrates sequencing saturation curve analysis for library completeness evaluation with pathway enrichment validation to confirm biological relevance, providing novel technical perspectives for host-pathogen interaction research. While the overall experimental framework (covering library construction, sequencing, data analysis, and functional validation) is generally sound, the following issues require improvement:

1. Please add some content regarding the application of Nanopore Sequencing Technology in quality control of cDNA libraries in the introduction section.
2. Some of the figure explanations are not clear enough. Please add some figure explanations more detailed alongside the figures, such as Figures 5 and 7-9.
3. The quality of RNA, efficiency of cDNA synthesis, and size distribution of inserted fragments in library construction are not clearly defined.
4. 12123 protein-coding genes were detected, comparative analysis with existing Y2H library studies should be supplemented to contextualize these findings.
5. A direct comparative analysis of gene detection performance between Nanopore Sequencing Technology and Illumina platforms using identical libraries is essential to substantiate the technical advantages of long-read sequencing.
6. The discussion should address inherent limitations of Nanopore Sequencing Technology technology, such as the coverage bias in high-GC or repetitive genomic regions, and the inability to distinguish alternative splicing isoforms.
7. The potential impact of undetected coding genes on Y2H false-negative rates should be systematically discussed.
